# The phosphatase PPM1A inhibits triple negative breast cancer growth by blocking cell cycle progression

**DOI:** 10.1038/s41523-019-0118-6

**Published:** 2019-07-26

**Authors:** Abhijit Mazumdar, William M. Tahaney, Lakshmi Reddy Bollu, Graham Poage, Jamal Hill, Yun Zhang, Gordon B. Mills, Powel H. Brown

**Affiliations:** 1 Department of Clinical Cancer Prevention, The University of Texas M.D. Anderson Cancer Center, Texas, USA; 2 Department of Molecular and Cellular Biology, Baylor College of Medicine, Houston, TX 77030 USA; 3 Biotheranostics, Inc, San Diego, CA 92121 USA; 4 Department of Cell, Developmental & Cancer Biology, Oregon Health & Science University, Oregon, USA

**Keywords:** Breast cancer, Predictive markers

## Abstract

Estrogen receptor (ER)-negative, progesterone receptor (PR)-negative and HER2-negative, or “triple negative,” breast cancer (TNBC) is a poor prognosis clinical subtype that occurs more frequently in younger women and is commonly treated with toxic chemotherapy. Effective targeted therapy for TNBC is urgently needed. Our previous studies have identified several kinases critical for TNBC growth. Since phosphatases regulate the function of kinase signaling pathways, we sought to identify critical growth-regulatory phosphatases that are expressed differentially in ER-negative, as compared to ER-positive, breast cancers. In this study, we examined the role of one of these differentially expressed phosphatases, the protein phosphatase Mg + 2/Mn + 2 dependent 1A (*PPM1A*) which is underexpressed in ER-negative breast cancer as compared to ER-positive breast cancers, in regulating TNBC growth. We found that PPM1A is deleted in ~40% of ER-negative breast cancers, and that induced expression of PPM1A suppresses in vitro and in vivo growth of TNBC cells. This study demonstrates that induction of PPM1A expression blocks the cell cycle and reduces CDK and Rb phosphorylation. These results suggest PPM1A is a crucial regulator of cell cycle progression in triple negative breast cancer. Our results also suggest that PPM1A loss should be explored as a predictive biomarker of CDK inhibitor sensitivity.

## Introduction

Breast cancer is one of the most commonly diagnosed cancers in women, and was the second-leading cause of cancer-related death in women in the United States in 2017.^1^ Most breast cancers are estrogen receptor (ER)-positive, which have a relatively good prognosis. Selective estrogen receptor modulators (e.g., tamoxifen) and aromatase inhibitors (e.g., anatrozole, letrozole, and exemestane) are currently used for the treatment of these ER-positive breast cancers either alone or in combination with chemotherapy.^2–6^ Alternatively, ER-negative HER2-positive breast cancers can be treated with anti-HER2 drugs, (e.g., trastuzumab and lapatinib^7–9^). However, limited targeted therapies are available for the most aggressive ER-negative breast cancers, “triple negative” breast cancers (TNBCs), named for their lack of expression of ER, progesterone receptor (PR), and Her2. While the relatively rare BRCA1/2 mutant population of TNBCs are treated with PARP inhibitors, the majority of TNBC tumors are currently treated with cytotoxic and non-specific chemotherapy.

We have previously conducted RNA microarray studies to identify potentially therapeutic targets of ER-negative breast cancers.^10–13^ These previous studies have identified several kinases critical for the growth of TNBC. We also previously showed that the phosphatase DUSP4, down-regulated in ER-negative breast cancers, regulates the growth of TNBC cells by inhibiting MAPK and other growth regulating pathways.^13^ We have also identified phosphatases that are overexpressed in TNBC when compared to ER-positive breast cancer, some of which are critical for the growth of TNBC cells. PTP4A3, one of the identified phosphatases, is a critical regulator of TNBC tumor growth in vivo.^12^ In the current study, we investigated the role of another key phosphatase that is differentially expressed in ER-negative tumors in regulating breast cancer survival and growth. PPM1A is one of the most differentially underexpressed phosphatases in ER-negative breast cancers. Using cBioPortal^14,15^ and the Molecular Taxonomy of Breast Cancer International Consortium (METABRIC) datasets,^16,17^ we determined that, of the top ten underexpressed phosphatases in ER-negative breast cancer, *PPM1A* (Protein Phosphatase Mg + 2/Mn + 2 Dependent) is the most frequently deleted phosphatases in ER-negative, compared to ER-positive, breast cancer.

PPM1A is a member of the protein phosphatase 2C family of Ser/Thr protein phosphatases.^18^ PPM1A has been shown to regulate TGF-beta/Smad^19–21^ and mitogen activated protein kinase^22^ cellular signaling pathways. PPM1A has been shown to regulate proliferation,^22^ cell invasion,^23^ and migration,^23^ but how PPM1A regulates these activities is not understood. Our results demonstrate PPM1A is deleted frequently in breast cancer, is underexpressed in TNBCs, and that overexpression of PPM1A reduces TNBC tumor growth. Our results also demonstrate phosphorylation of CDKs and Rb is reduced by PPM1A overexpression and provide a molecular basis for the observed growth suppression induced by PPM1A expression. Overall, this study demonstrates PPM1A is frequently deleted in ER-negative breast cancers, and that loss of PPM1A promotes the growth of TNBCs, suggesting that PPM1A is an important tumor suppressive gene in these aggressive breast cancers.

## Results

### Expression of PPM1A in breast tumors

To identify phosphatases that are differentially expressed in ER-negative breast cancers, we previously compared RNA levels in ER-positive and ER-negative human breast cancer samples using RNA profiling.^12,13^ Through these analyses, we identified a set of phosphatases that are differentially expressed in ER-negative as compared to ER-positive breast cancers. In the current study, we focused on the PPM1A phosphatase that is underexpressed in ER-negative breast cancers.

**Fig. 1:**
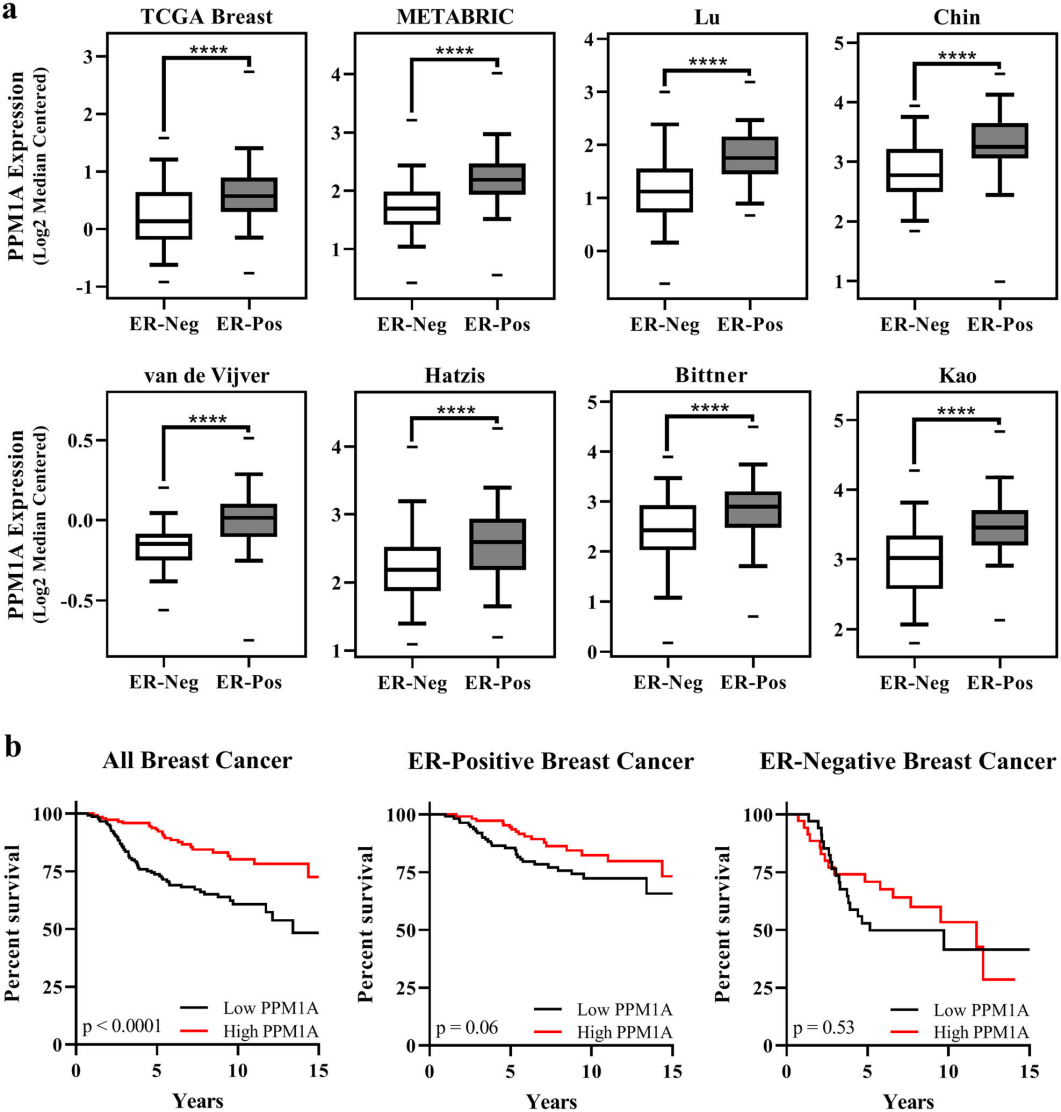
PPM1A is underexpressed in ER-negative breast cancer and correlates with poor survival. **a** PPM1A is underexpressed in ER-negative breast cancer compared to ER-positive breast cancer in eight publically available datasets. Center lines show median, whiskers represent 95% confidence intervals, and dashes indicate maximum and minimum values. *p*-values were calculated using Student’s *t*-test (****p *<* 0.0001). **b** Low PPM1A expression correlates with poor survival. Kaplan–Meier curves were generated from the van de Vijver dataset, showing overall survival of all breast cancer (*n* = 295), ER-positive (*n* = 266), and ER-negative (*n* = 69) patients. High and low expression groups were dichotomized by median PPM1A expression in each group. Curve comparisons of stratified groups were calculated using the Mantel-Cox log-rank test, with *p* < 0.05 being considered statistically significant

We first conducted an examination of *PPM1A* expression across several publicly available breast cancer microarray datasets.^16,24–30^ Details of these datasets are described in “Methods” and are listed in Mazumdar et al.^31^ As shown in Fig. 1a, PPM1A is underexpressed in ER-negative tumors as compared to ER-positive tumors in eight individual human breast cancer data sets.

### High PPM1A expression has a favorable clinical outcome

Since *PPM1A* is underexpressed in ER-negative breast cancer, we next examined whether there is an association between *PPM1A* expression and patient survival. We performed survival analyses in breast tumor datasets that included overall survival. Subjects in the Van de Vijver dataset^24^ (*n* = 295) were grouped based on *PPM1A* expression with high and low *PPM1A* groups (defined as expression above or below the median). Individuals with low *PPM1A*-expressing tumors have significantly lower overall survival rates compared to subjects with high *PPM1A*-expressing tumors (*p* < 0.0001) (Fig. 1b). Analysis of the women with ER-positive tumors yielded similar poor survival with PPM1A low tumors (*p* = 0.06). The difference in ER-negative tumors was not statistically significant (*p* = 0.53), likely because patients were dichotomized into high and low groups by median PPM1A expression, and the “high PPM1A” patients with ER-negative tumors exhibited a lower level of PPM1A than the “high PPM1A” patients with ER-positive tumors (Fig. 1b). Furthermore, Cox proportional hazards regression analysis of the Van de Vijver dataset^24^ (adjusted for both ER and node status) showed that *PPM1A* expression is an independent predictor of survival (HR = 0.55; *p* < 0.02) (data not shown).

### PPM1A is preferentially deleted in ER-negative, but not ER-positive breast cancer

**Fig. 2:**
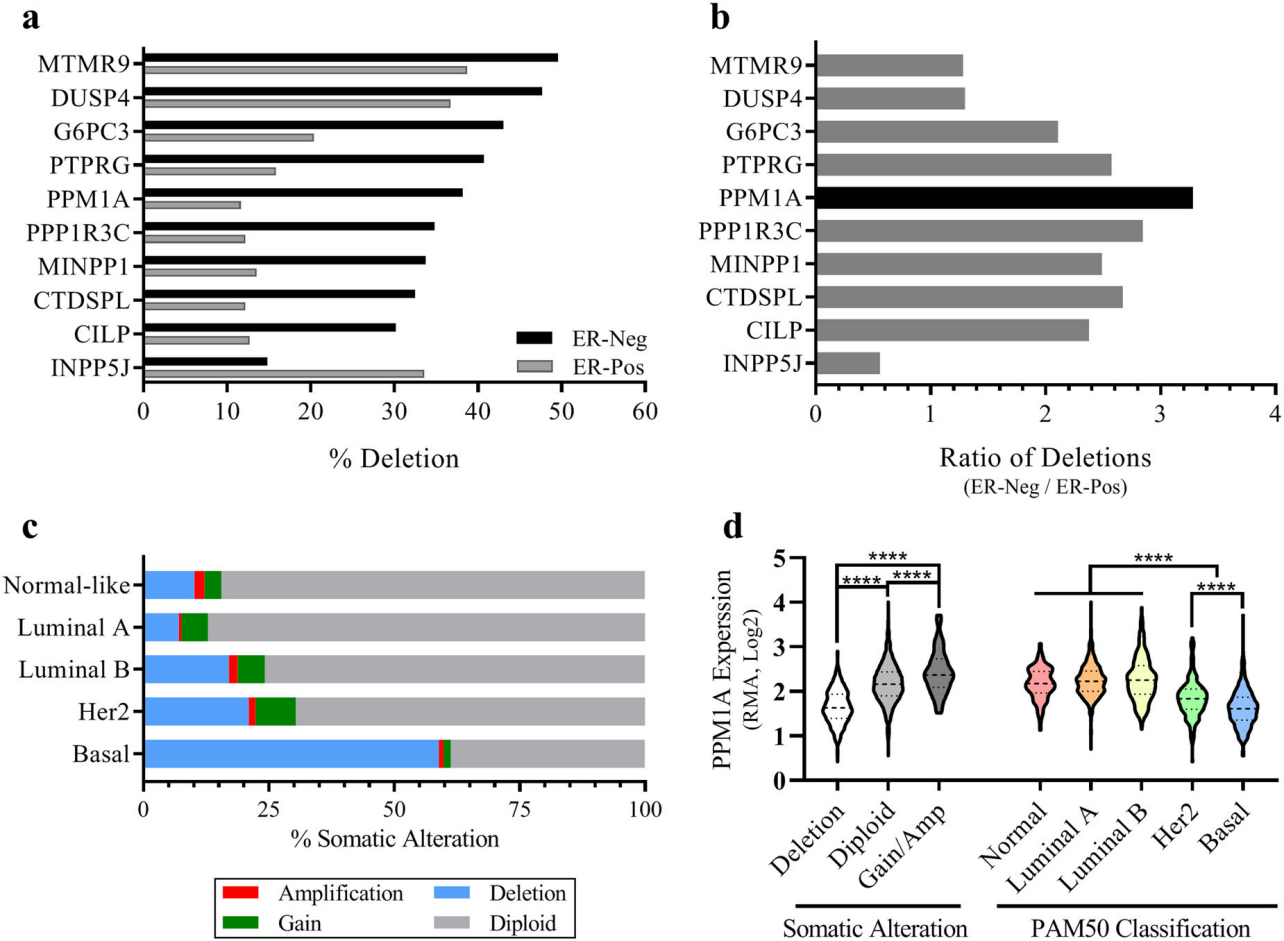
PPM1A is preferentially deleted in ER-negative Breast Cancer. **a** Comparison of phosphatase deletions in ER-negative and ER-positive breast cancer in the METABRIC dataset. Both heterozygous and homozygous deletions are collectively termed “deletion.” **b** PPM1A is preferentially deleted in ER-negative, compared to ER-positive, breast cancer. **c** PPM1A is most frequently deleted in the basal Pam50 subtype. **d** PPM1A mRNA expression correlates with somatic alteration and PAM50 classification. The violin plot shows median (dashed line) and quartiles (dotted lines), and *p*-values were calculated using Student’s *t*-test (****p *<* 0.0001)

To investigate why the mRNA level of PPM1A is low in ER-negative breast cancer, we profiled the top ten underexpressed phosphatases for allelic deletions using cBioPortal and the METABRIC dataset. Of these ten phosphatases, nine showed preferential deletion in ER-negative breast cancer, compared to ER-positive (Fig. 2a). While nine phosphatases demonstrated more frequent deletion in ER-negative breast cancer than ER-positive, the difference between these two subtypes of breast cancer varied. By comparing the frequency of phosphatase deletion between ER-negative and ER-positive breast cancer, we found PPM1A to be the most preferentially deleted in ER-negative breast cancer (Fig. 2b). To examine whether PPM1A deletion was more often seen in a particular breast cancer subtype, we compared the somatic alteration of PPM1A across different breast cancer subsets defined by Pam50 gene signatures.^32^ PPM1A deletion was seen most commonly in basal breast cancers, at a rate of approximately 60%, while a rate of only 10–20% was observed in other breast cancer subtypes. On the other hand, amplification and chromosomal gains were seen more commonly in normal like, luminal and Her2-enriched subtypes (Fig. 2c). We next sought to determine the effect of PPM1A somatic alteration on PPM1A expression. We compared the PPM1A expression of METABRIC samples with PPM1A deletions, gains and amplifications, and diploid copy numbers, and discovered that samples with PPM1A deletions had a statistically lower PPM1A expression than diploid samples (*p* < 0.0001). Analogously, samples with a gain or amplification in PPM1A had a statistically significant increase in PPM1A expression (*p* < 0.0001) (Fig. 2d). Finally, we examined PPM1A expression among the PAM50 classification, and determined that the predominantly ER-negative Her2 and basal subtypes exhibited significantly lower PPM1A expression. The basal subtype, found to have the highest percentage of PPM1A deleted samples, also displayed the lowest PPM1A expression, significantly lower than the Her2 samples (*p* < 0.0001) (Fig. 2d). Taken together, these results demonstrate that PPM1A is preferentially deleted in ER-negative breast cancer, and this correlates with lower PPM1A mRNA expression.

### Inducible expression of PPM1A inhibits ER-negative but not ER-positive breast cancer cell growth in vitro

**Fig. 3:**
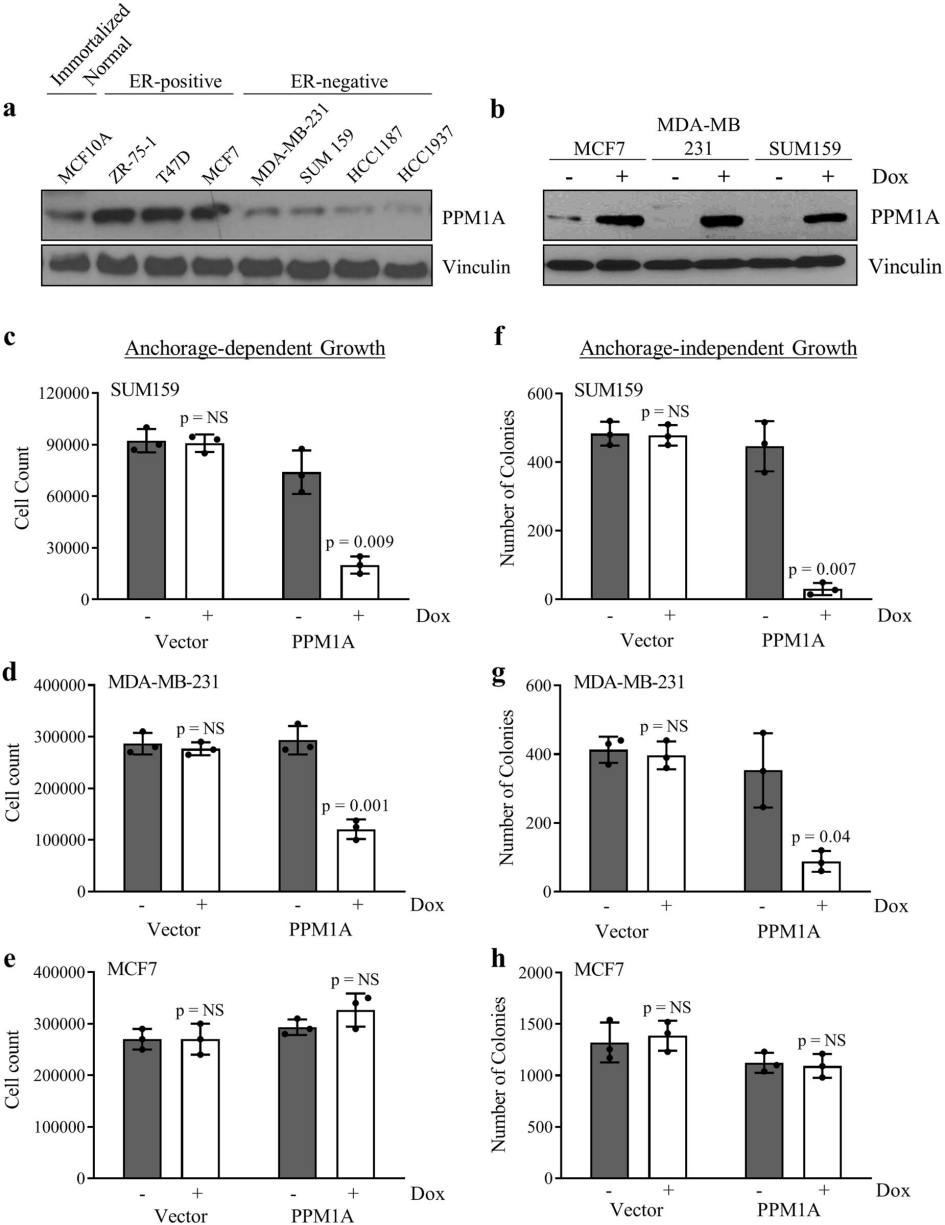
Induced expression of PPM1A inhibits ER-negative but not ER-positive growth in vitro. **a** PPM1A and Vinculin protein expression in multiple breast cancer lines cell. **b** Doxycycline-induced protein expression of PPM1A and vinculin in MCF7, MDA-MB-231 and SUM159 breast cancer cells. **c**–**e** Analysis of breast cancer cell line proliferation upon PPM1A expression. **f**–**h** Analysis of breast cancer cell line anchorage-independent colony formation upon PPM1A expression. All error bars represent SD, and *n* = 3. *p*-values were calculated using Student’s *t*-test

To investigate the role of PPM1A in regulating breast cancer survival and growth, we first compared PPM1A protein levels in ER-positive and ER-negative breast cancer cell lines, and an immortalized normal breast cell line. As shown in Fig. 3a, immortalized normal breast and ER-positive breast cancer cell lines express higher levels of PPM1A than the ER-negative cell lines. To study the role of PPM1A in the regulation of breast cancer cell growth, we cloned *PPM1A* cDNA into a tetracycline (Tet)-inducible vector (pTIPZ). pTIPZ-PPM1A or pTIPZ-vector containing lentiviral particles were infected, from which stable pools of two ER-negative cell lines (SUM159 and MDA-MB-231), and one ER-positive cell line (MCF7), were generated through puromycin selection for doxycycline-inducible PPM1A expression. After 4 days of induction with doxycycline, PPM1A expression was determined with western blotting using an anti-PPM1A antibody. Our results demonstrate that PPM1A expression was significantly induced in the breast cancer cell lines after 4 days of doxycycline treatment (Fig. 3b).

We next used cell counting to determine the effect of PPM1A on breast cancer cell growth with and without doxycycline treatment. Our results indicate induced expression of PPM1A can strongly suppress cell growth of the PPM1A-transfected ER-negative cell lines tested (SUM159: 73% repression; MDA-MB-231: 59% repression), but does not suppress growth in PPM1A-transfected ER-positive (MCF7) breast cancer cells (Fig. 3c–e, respectively). Additionally, cell growth rate was not significantly altered with doxycycline treatment in any of the vector-transfected cells. Taken together, these results demonstrate that induction of PPM1A inhibits proliferation of ER-negative, but not ER-positive, breast cancer cells.

We also determined whether PPM1A regulates anchorage-independent growth of breast cancer cells using the aforementioned doxycycline-inducible cell lines. Our results show that induced PPM1A expression markedly suppresses anchorage-independent growth of both PPM1A-transfected ER-negative cells tested (SUM159: 93% suppression; MDA-MB-231: 75% suppression), but not of the PPM1A-transfected ER-positive (MCF7) cells (Fig. 3f–h, respectively). Treatment of vector clones with doxycycline showed no alteration in anchorage-independent. These results further demonstrated that induction of PPM1A inhibits growth of ER-negative, but not ER-positive, breast cancer cells.

### Induction of PPM1A inhibits ER-negative breast cancer growth in vivo

**Fig. 4:**
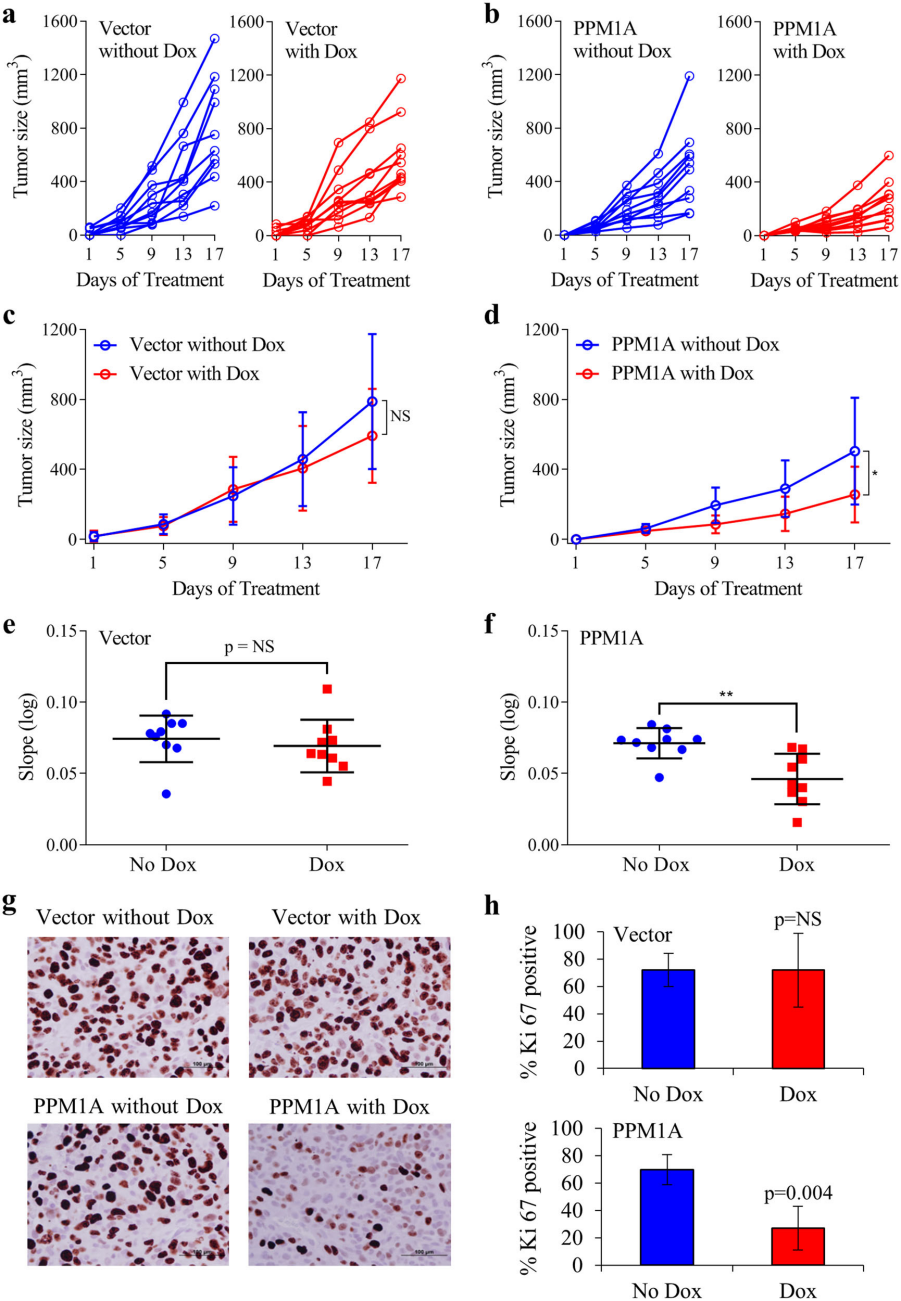
Induction of PPM1A expression inhibits growth and proliferation of ER-negative breast cancer cells in vivo. **a**, **b** In vivo growth of individual MDA-MB-231-PPM1A and Vector control xenografts, with and without dox treatment. **c**, **d** Comparison between average tumor size with and without dox treatment for MDA-MB-231-PPM1A and vector control xenografts. Comparisons between average tumor growth was performed with Two-way Repeated Measures ANOVA (*p < 0.05). **e**, **f** Calculated slope of MDA-MB-231-vector and PPM1A clone xenografts, with indicated Student’s *t*-test values (**p < 0.01). **g** Representative images for Ki67 staining of MDA-MB-231 xenograft sections. **h** Quantitation of Ki67 xenograft sections. All error bars represent SD, and *n* = 10. *p*-values were calculated using Student’s *t*-test

We then investigated whether PPM1A expression can inhibit in vivo growth of TNBCs using nude mouse xenografts. We injected MDA-MB-231-PPM1A and Vector control cells into the mammary fat pad. After injected cell lines formed tumors of approximately 50 mm^3^ in volume, the mice were randomized into two groups receiving doxycycline-treated or non-doxycycline-treated water. The growth rate of the MDA-MB-231-vector clone was unaffected by doxycycline treatment (Fig. 4a). However, strong growth suppression of tumors was associated with doxycycline-induced PPM1A expression in MDA-MB-231 xenografts as compared to no doxycycline treatment (Fig. 4b). In comparing average tumor size, there was no statistical difference between MDA-MB-231-vector clones with and without dox, but there was statistically different between MDA-MB-231-PPM1A clones with and without dox treatment (*p* < 0.05) (Fig. 4c, d). Additionally, there was no difference in growth rate (slope) observed in the MDA-MB-231-vector control clones (Fig. 4e). However, there was a significant difference in the MDA-MB-231-PPM1A clone growth rates, dependent on the presence and absence of doxycycline (*p* < 0.01) (Fig. 4f). As shown in Fig. 4g, h, Ki67 expression in these MDA-MB-231 tumors, measured by IHC, was reduced significantly in the PPM1A-induced group upon doxycycline treatment (*p* = 0.004). Consistent with our in vitro studies, these findings demonstrate the induction of PPM1A inhibits ER-negative breast tumor growth in vivo.

### PPM1A induces blockade of cell cycle progression

**Fig. 5:**
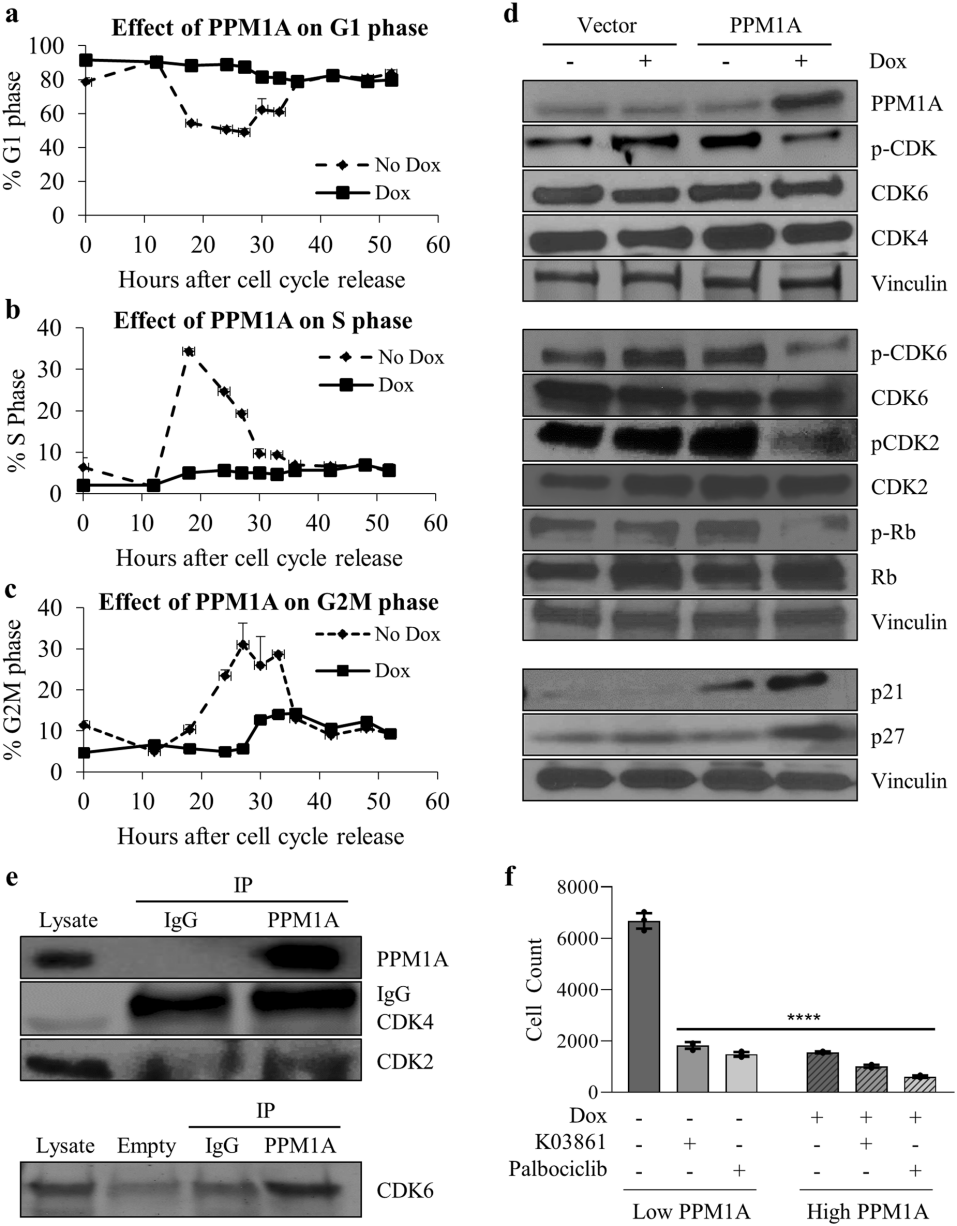
Cell cycle changes and signaling pathways altered upon induced expression of PPM1A induction. **a** Effect of PPM1A on G1 (No Dox = No PPM1A overexpression; Dox = PPM1A overexpression, **b** on S phase, and **c** on G2-M phase. Error bars represent SD, and *n* = 3. **d** Western blot analysis of PPM1A and changes in p-CDK, total CDK6, total CDK4, p-CDK6, p-CDK2, total CDK2, p-RP, total RB, p21, p27, and vinculin. **e** PPM1A interacts with CDK6 and CDK2, but not with CDK4, as detected by immunoprecipitation/western blot analysis. **f** Inhibition of CDK function using the CDK2 inhibitor K03861 and CDK4/6 inhibitor Palbociclib mimics the growth suppression of PPM1A overexpression on triple negative breast cancer cells. Error bars represent SD, and *n* = 4. *p*-values were calculated using Student’s *t*-test (****p *<* 0.0001)

We next investigated the effect of PPM1A on the cell cycle using flow cytometry analysis and PI staining to determine the mechanism by which PPM1A reduces proliferation. Dox-inducible SUM159-PPM1A cells were synchronized with lovastatin, then released from G0/G1 blockade using mevalonate and collected and fixed overnight. The cells were then stained and subjected to cell cycle analysis. Our results, reflecting three independent experiments, show that induced expression of PPM1A arrests breast cancer cells in G0/G1 phase and prevents S-phase entry. Significant differences in G1 phase between Vector control and PPM1A-expressing cells were observed as early as 18 h. Vector control cells enter into the cell cycle normally; however, PPM1A-expressing cells remain in the G0/G1 phase of the cell cycle (Fig. 5a) and do not enter S-phase (Fig. 5b) or the G2/M phase (Fig. 5c). Our cell cycle analyses suggested that PPM1A-induced blockade of cell cycle progression is one of the mechanisms inhibiting growth of PPM1A-expressing ER-negative breast cancer cells.

### Induced expression of PPM1A inhibits the phosphorylation of cell cycle regulating proteins

To investigate the cause of this cell cycle blockade, we analyzed various cells cycle markers including phospho-panCDK, total and phospho-CDK6, total CDK4, total and phospho-CDK2, total and phospho-Rb, p21, and p27. Our results indicate that induced expression of PPM1A reduces phosphorylation of CDK (pan), CDK2, CDK6, and Rb without altering corresponding total proteins (Fig. 5d). Induced expression of PPM1A also results in the induction of the cell cycle regulatory proteins p21 and p27 (Fig. 5d). To elucidate mechanisms contributing to the inhibition of phosphorylation of CDK upon PPM1A induction, we performed immunoprecipitation and western blot analysis using CDK2, CDK4, and CDK6 antibodies. Our results indicate that CDK2 and CDK6 directly interact with PPM1A (Fig. 5e). There was no interaction between PPM1A and CDK4. Our results suggest that PPM1A interacts directly with CDK2 and CDK6 to dephosphorylate these proteins, which then blocks entry into S-phase and G2-phase.

### Inhibition of CDK function suppress triple negative breast cancer cells growth

To investigate whether the expression of PPM1A alters the growth inhibitory effect of CDK inhibitors, we measured Dox-inducible SUM159-PPM1A growth in presence and absence of Dox (to induce PPM1A), Palbociclib (CDK4/6 inhibitor), and K03861 (CDK2 inhibitor). Our data demonstrates that both Palbociclib and K03861 strongly suppresses the growth of SUM159 with low PPM1A (without Dox condition) (Fig. 5f). In presence of PPM1A overexpression (with Dox condition), the inhibitory effect of these CDK inhibitors (K03861and Palbociclib) is blunted. Taken collectively, these results suggest that PPM1A exerts its growth inhibitory through reduction of phosphorylation of CDK6 and CDK2 cyclin-dependent kinases.

## Discussion

In this study we investigated the role of PPM1A, a phosphatase underexpressed in ER-negative as compared to ER-positive breast cancers, in the regulation of breast cancer growth. Our results show that tumors with low PPM1A have a poor clinical outcome. We also found that among the top ten most deleted phosphatases, PPM1A is the most frequently deleted phosphatase in ER-negative breast cancers as compared to ER-positive breast cancers, and that this deletion correlates with a significant decrease in PPM1A mRNA expression. We also demonstrated that induced expression of PPM1A suppresses both in vitro and in vivo growth of ER-negative breast cancer cells. Mechanistic studies showed that induced PPM1A blocks the cell cycle of ER-negative cells at the G1/S and inhibits CDK and Rb phosphorylation, which results in the arrest of breast cancer cell growth. Inhibition of CDK4/6 and CDK2 function using biochemical inhibitors suppresses the growth of triple negative breast cancer cells with low PPM1A, suggesting PPM1A as a biomarker to predict sensitivity to CDK inhibitors for more effective treatment of triple negative breast cancer.

**Fig. 6:**
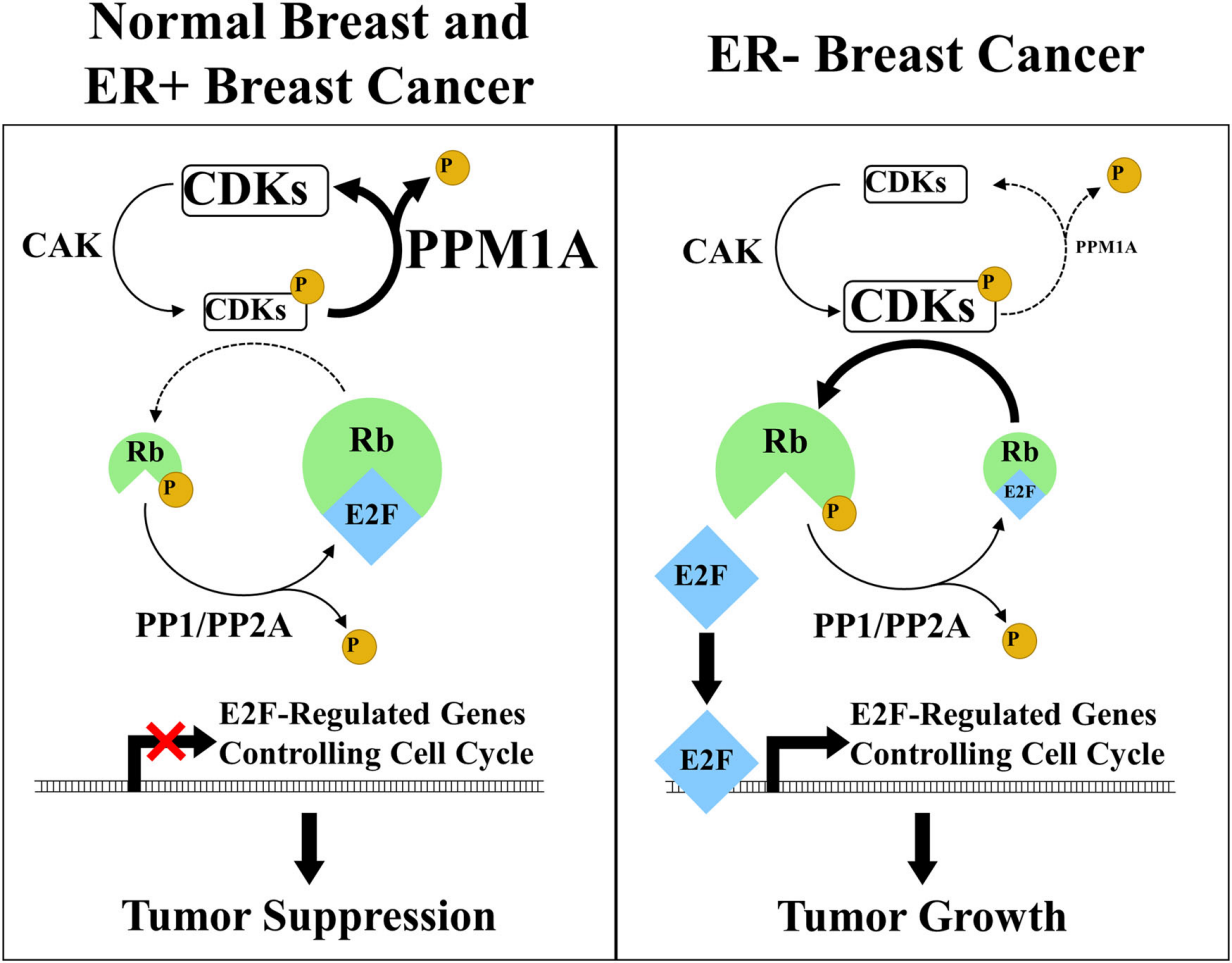
Proposed schematic presentation of PPM1A regulated growth suppression in ER-negative and ER-positive breast cancers through CDK inhibition

Based on our findings, we propose the model depicted in Fig. 6. In normal breast cells and ER-positive breast cancers, PPM1A expression is high. This causes CDKs to be dephosphorylated, thus minimizing the phosphorylation of RB, and allowing unphosphorylated RB to bind E2F, making E2F unavailable to bind DNA and unable to stimulate the transcription of E2F-regulated genes. This results in reduced growth, and in normal breast cells, suppression of tumorigenesis. However, in ER-negative cells where PPM1A expression is low, CDKs are not dephosphorylated, and the phosphorylated form of CDK6 phosphorylates RB, which causes the release of E2F, allowing it to bind DNA and induce transcription of E2F-regulated genes, ultimately stimulating tumor growth (Fig. 6). In addition, PPM1A reduces the phosphorylation of CDK2 and thereby additionally regulates cellular division at S-phase.

Previous studies in yeast support the proposal that PPM1A inactivates Cdk2 and CDK6.^33,34^ Our results in ER-negative breast cancer models show that PPM1A dephosphorylates CDKs and that it interacts directly with CDK6 and CDK2. Prolonged phosphorylation of CDK6, an important regulator of G0/G1 phase leads to unregulated cell cycle progression and, subsequently, increased growth. Our finding suggests that by reducing phosphorylation of CDK6 and CDK2, PPM1A suppresses the growth of ER-negative breast cancers.

PPM1A is located on chromosome 14, cytoband 14q23.1. Bergamaschi et al. showed copy number alterations (CNAs) in basal-like breast cancer subtype tumors at 14q23.1, along with other CNAs.^35^ Therefore, our bioinformatic data showing PPM1A deletion in the basal subtype are in agreement with the CNA analysis by Bergamaschi.^35^ Several other publically available breast cancer datasets, accessed through Oncomine, also show similarly reduced expression of PPM1A in ER-negative and TNBC tumors compared to ER-positive tumors. PPM1A downregulation has been observed in many other cancers including prostate, bladder, ovarian, and liver.^36–39^ In prostate cancer, PPM1A acts as a RelA phosphatase and has tumor suppressor like activity.^36^ In subcutaneous xenotransplantation and lung metastasis models of bladder cancer, overexpression of PPM1A significantly reduces the growth and metastasis of bladder cancer through the regulation of TGF-beta signaling pathways suggesting that PPM1A is a candidate tumor suppressor.^37^ In a study of a several tumor types, loss of PPM1A induces phosphorylation of BAD and inhibits apoptotic pathways, also suggesting a tumor suppressor role.^38^

Our results show that overexpression of PPM1A in TNBC cells profoundly suppresses cellular growth particularly under 3D conditions and in vivo. However, there was no effect of PPM1A overexpression on ER-positive cells growth (MCF7). Lammers et al.^40^ also showed that knockdown of PPM1A has no effect on cell proliferation in MCF7 cells. These results suggest that loss or induced expression of PPM1A does not affect the growth of ER-positive breast cancer cells, as it does in ER-negative breast cancer cells. The ability of ER-positive breast cancers to grow, even in the presence of high levels of PPM1A suggests that in these cancers rely on other pathways, such as those stimulated by the estrogen and the estrogen receptor, support unregulated growth in ER-positive breast cancers.

In summary, we identified the PPM1A phosphatase to be differentially expressed in ER-negative breast cancers compared to ER-positive breast cancers. We demonstrated that induced expression of PPM1A in ER-negative breast cancer cells inhibits their growth in vitro and in vivo. We also showed that PPM1A suppresses cell growth by inhibiting CDK phosphorylation, inducing cell cycle inhibitors p21 and p27, and ultimately causing a cell cycle block. These studies support further study of PPM1A as a biomarker to predict sensitivity to CDK inhibitors for more effective treatment of triple negative breast cancer.

## Methods

### Bioinformatics analysis

This tumor set analysis was previously described.^10^ Analysis of PPM1A RNA expression was performed with the Oncomine platform^41^ and publicly available datasets.^16,24–30^ Analysis of allelic deletion and somatic alteration of breast cancer tumors was completed using cBioPortal^14,15^ and the METABRIC dataset.^16,17^ Ratios of somatic phosphatase deletions were calculated by dividing percent allelic deletion of ER-negative breast cancer by ER-positive breast cancer.

### Cell lines and cell culture methods

All cell lines were obtained from the American Type Culture Collection (ATCC, Manassas, VA). MCF7, ZR-75-1, T47D, and MDA-MB-231 breast cancer cell lines were cultured in DMEM and supplemented with 10% regular and non-charcoal stripped fetal bovine serum, 100 mg/ml streptomycin, and 100 U/ml penicillin. HCC1187 and HCC1937 were cultured in RPMI-1640 and supplemented with 10% regular and non-charcoal stripped fetal bovine serum, 100 mg/ml streptomycin, and 100 U/ml penicillin. The MCF10A cell line was maintained in DMEM/F12, supplemented with 5% horse serum, 20 ng/mL EGF, 500 µg/mL hydrocortisone, 100 ng/mL Cholera toxin, 10 µg/mL insulin, 100 mg/ml streptomycin, and 100 U/ml penicillin. The SUM159 cell line was maintained in Ham’s F12 medium supplemented with 5% FBS, 1 µg/ml hydrocortisone, 5 µg/ml insulin, 100 mg/ml streptomycin, and 100 U/ml penicillin.

Cell lines were tested for mycoplasma by PCR and their identity was confirmed using short tandem repeat (STR) DNA fingerprinting using the AmpFLSTR Identifiler PCR Amplification Kit (cat 4322288, Life Technologies, Foster City, CA), according to manufacturer instructions. STR profiles were compared to: (1) the MD Anderson fingerprint database, (2) known ATCC fingerprints (ATCC.org), and (3) the Cell Line Integrated Molecular Authentication database (CLIMA) version 2.0.201406 (http://bioinformatics.hsanmartino.it/clima/).^42^

### Reagents and antibodies

PPM1A (PP2C-α), total and phospho-CDK2, total CDK4, total CDK6, phospho-Rb, p21, and p27 antibodies were obtained from Cell Signaling Technology, Inc. (Danvers, MA). The phospho-pan CDK antibody was obtained from Santa Cruz Bioscience (Dallas, TX). The phospho-CDK6 antibody was obtained from Thermo Scientific (Waltham, MA). The total Rb antibody was obtained from BD Transduction Laboratories (Lexington, KY). The vinculin antibody was obtained from Sigma-Aldrich Corp. (St. Louis, MO). Anti-mouse and anti-rabbit HRP secondary antibodies were purchased from GE Healthcare Bio-Sciences Corp. (Piscataway, NJ). Anti-mouse and anti-rabbit IRDye secondary antibodies were purchased from LI-COR Biosciences (Lincoln, NE). CDK inhibitors Palbociclib (CDK4/6) and K03861 (CDK2) were purchased from Selleck Chemical (Houston, TX).

### Viral vectors and cDNAs

The PPM1A ORF clone was purchased from Open Biosystems (Huntsville, AL) and subsequently cloned into the tetracycline (Tet)-inducible vector pTIPZ using the Gateway LR Clonase reaction. Resulting constructs were verified by sequencing and restriction enzyme digestion. Lentiviral vectors were prepared by standard methods of cotransfection of viral helper plasmids (GAG, POL, TAT, and VSV-G) in 293T cells with a media change 24 h post-transfection. Media was harvested at 24, 48, and 72 h post-change, filtered through a 0.45 µm MEC filter, and viral vectors were concentrated by high speed centrifugation. Concentrated virus was then aliquoted and stored at −80 °C. Viruses were tittered using serial dilution and cells were infected at a multiplicity of infection (MOI) of ~1. Cell lines stably expressing inducible cDNAs were generated through lentiviral infection using a pTIPZ lentiviral expression system supplemented with 4 μg/ml polybrene, followed by selection using puromycin at 48 hours post-infection. All resulting pTIPZ stable cell lines were subsequently maintained in media with Tet System Approved FBS (Clontech Laboratories Inc., Mountain View, CA). Within 4–5 days of infection, TIPZ-infected cells were expanded, induced with doxycycline (Dox), and either tested for PPM1A overexpression or used in assays.

### Cell proliferation assays

Cellular proliferation was measured by plating 4000 cells/well on 24-well plates and counting viable cells at indicated time points using trypan blue exclusion and the Invitrogen Countess automated cell counter (Thermo Scientific, Waltham, MA). Experimental data points were performed in quadruplicate, and results were reported as average number ± SD. Cell proliferation in presence and absence of CDK2 and CDK4/6 inhibitors was measured by plating 1000 SUM159-PPM1A cells/well in 96 well plates. After 24 h, cells were treated with CDK inhibitors (K03861 and Palbociclib) at 10 μΜ. Five days after drug treatment, cells were fixed with 4% paraformaldehyde in PBS. Nuclei were then stained with DAPI and imaged with the MetaXpress microscope (Molecular Devices, San Jose, CA). Cell nuclei were segmented then counted by defining pixel intensity over background and object size, using the algorithm of the MetaXpress image analysis software. Experimental data points were performed in quadruplicate, and results were reported as average cell count ± SD.

### Anchorage-independent growth assays

Anchorage-independent soft agar growth assays were performed as previously described.^43^ Briefly, 20,000 cells/well were plated on 6-well plates in 0.35% soft agar (SeaPlaque Soft Agarose, FMC Corporation, Rockland, ME) layered over a base of 0.7% soft agar. Plated cells were then allowed to grow in cell-specific ATCC recommended media for 1–5 weeks, with duration dependent upon cell type. Colonies were fed weekly with media and measured using a GelCount Soft Agar Colony Counter (Oxford Optronix, Oxford, UK). Colonies between 50 μm and 250 μm in diameter were selected and scored. Experimental data points were performed in triplicate, and results were reported as average number ± SD.

### Cell cycle assays

To measure cell cycle distribution, the Tet-inducible stable cell line SUM159 was treated for 4 days with or without doxycycline (2 μg/ml) to induce PPM1A gene expression. Following this, cells were synchronized using lovastatin (20 μM) for 36 hours followed by cell cycle release with mevalonate (2 mM). Cells were then harvested at 0–52 h and fixed overnight at −20^0^C with 20% ethanol. Cells were stained with propidium iodide (PI) (1 μg/ml) in 0.1% Triton X-100 and RNase in PBS, then analyzed with a FACSCalibur Flow Cytometer (BD Biosciences, Franklin Lakes, NJ).

### Western blot analysis

Western blot analyses were performed as previously described.^13^ Briefly, 30–50 µg total protein extract was run on a 10% SDS-PAGE gel, then transferred to a nitrocellulose membrane (GE Healthcare, Piscataway, NJ). Membranes were blocked in PBST using 5% non-fat milk or 5% BSA, then incubated overnight at 4 °C with PPM1A, Total and phospho-CDK2, total CDK4, total CDK6, phospho-RB, p21, and p27 antibodies (all from Cell Signaling Technology Inc., Danvers, MA, 1:1000), phospho-pan CDK antibody (Santa Cruz Bioscience, Dallas, TX, 1:500), phospho-CDK6 antibody (Thermo Scientific, Waltham, MA, 1:1000), total Rb antibody (BD Biosciences, San Jose, CA, 1:1000), or anti-Vinculin antibody (Sigma-Aldrich Corp., St. Louis, MO, 1:5000). Membranes were then washed and incubated with secondary anti-rabbit or anti-mouse antibody conjugated with IRDye (LI-COR, Lincoln, NE, 1:10,000) or anti-mouse/anti-rabbit HRP conjugated (GE Healthcare Bio-Sciences Corp., Piscataway, NJ, 1:3000). All blots shown derive from the same experiment, and were processed in parallel.

### Immunoprecipitation

Protein lysate was immunoprecipitated with PPM1A-specific antibody (Cell Signaling Technology Inc., Danvers, MA, 1 µg/mg protein lysate) and isotype matched IgG (Santa Cruz Biotechnology, Dallas, TX, 1 µg/mg protein lysate) at 4 °C overnight. Prior to the addition of antibody, the protein lysate was clarified with Protein A/G-Sepharose beads (Santa Cruz Biotechnology, Dallas, TX) to remove nonspecific binding. Protein A/G-Sepharose beads were then added to the lysate for an hour to isolate the antibody-bound complexes. The antibody-bound protein was eluted and separated on a 4–20% gradient SDS-PAGE gel. CDK6, CDK4, and CDK2 proteins were then detected with immunoblotting.

### Mouse experiments

Experiments using nude mice (The Jackson Laboratory, Bar Harbor, ME) were performed in accordance with M.D. Anderson Institutional Animal Care and Use Committee (IACUC)-approved protocols. MDA-MB-231-PPM1A and Vector control cells were injected into the mammary fat pads of nude mice (1.5 × 10^6^ per animal in 100 μl PBS) and measured at indicated time points. After tumors developed and reached a size of 40–50 mm^3^, mice were randomized into groups to receive doxycycline-containing (200 µg/ml) or doxycycline-free 30% sucrose-water to induce PPM1A expression. Tumor sizes were measured twice weekly with digital calipers. Volumes were calculated using the formula v = (width^2^ × length)/2. Tumor growth rates of different groups were calculated and statistically analyzed. Mice were sacrificed when tumors reached 1500 mm^3^. Comparisons between average tumor size, +/− dox, were performed with the Two-way Repeated Measures ANOVA (*p < 0.05). In vivo tumor growth was approximately exponential but exhibited variation between animals. Therefore, individual growth rates were estimated by calculating linear regression of log-transformed tumor volumes over time and then compared using Student’s *t*-test to compare tumor growth rates between doxycycline-treated and doxycycline-untreated animals. Growth rates were summarized by mean values and 95% confidence intervals (CIs).

### Immunohistochemistry

Tumor samples were fixed in 4% paraformaldehyde followed by embedding in paraffin. Tissue sections were mounted onto slides, then processed for Hematoxylin-eosin (H&E) staining or immunohistochemical (IHC) staining. H&E staining started by deparaffinizing 4 µm tissue sections in xylene. Sections were rehydrated in ethanol and water, followed by a 7 min incubation in hematoxylin. Samples were destained in running water, then fixed with acidified alcohol and ammonia. Finally, slides were incubated in eosin for 2 min, rinsed in alcohol and xylene, and then mounted for evaluation.

For IHC studies, 4 µm tissue sections were mounted on slides, which were then deparaffinized. Endogenous peroxidase was blocked in a 3% hydrogen peroxide buffer. Slides were then rinsed in PBS, with nonspecific binding being blocked with 10% albumin. Samples were incubated with the primary antibody Lab Vision anti-Ki67 (Thermo Scientific, Waltham, MA, prediluted) overnight at 4 °C. Following this, samples were incubated with biotinylated anti-rabbit antibody (Vector Laboratories, Inc., Burlingame, CA, 1:100) for 30 min. Peroxidase activity was visualized with the Vector NovaRED Substrate Kit (PK-6101, Vector Laboratories, Inc., Burlingame, CA) and the AEC Peroxidase Substrate Kit, 3-amino-9-ethylcarbazole (SK-4200, Vector Laboratories, Inc., Burlingame, CA). The slides were finally counterstained with hematoxylin for 30 s and mounted with cover slips.

### Statistical analyses

All values are expressed as average +/−standard deviation. Two-tailed Student’s *t*-test was used to access if the difference between the various experimental groups were significant. A *p*-value of less than 0.05 was considered to represent statistical significance.

### Reporting summary

Further information on experimental design is available in the Nature Research Reporting Summary linked to this paper.

## Supplementary information

Western Blot Data

Reporting Summary Checklist

## Data Availability

The datasets for this paper are listed in Mazumdar et al.^31^ mRNA expression data from eight publicly available datasets and the overall survival data of the van de Vijver dataset were accessed and analyzed through the bioinformatics portal Oncomine (www.oncomine.org) and support Fig. 1 in this article. The publicly available datasets as listed in Oncomine are: TCGA Breast, Curtis Breast, Lu Breast, Chin Breast, van de Vijver Breast, Hatzis Breast, Bittner Breast and Kao Breast. Allelic deletion and somatic copy number alterations of breast cancer tumors of the METABRIC dataset (Curtis Breast in Oncomine) were accessed through cBioPortal (https://identifiers.org/cbioportal:brca_metabric) and support Fig. 2 of the published article. The raw genomic data of the above datasets are also accessible from various repositories: TCGA dataset, available at NCBI dbGAP (https://identifiers.org/dbgap:phs000178.v10.p8), Curtis Breast dataset, available at the European Genome-phenome Archive, EGA (study accession ID: EGAS00000000083), Chin Breast dataset, available at Array Express (https://identifiers.org/arrayexpress:E-TABM-158), Van de Vijver Breast dataset, available at Computational Cancer Biology, Netherlands Cancer Institute (http://ccb.nki.nl/data/, A gene-expression signature as a predictor of survival in breast cancer, dataset: Genome-Wide Gene Expression Data for 295 Samples. The Lu Breast (https://identifiers.org/geo:GSE5460), Hatzis Breast (https://identifiers.org/geo:GSE25066), Bittner Breast (https://identifiers.org/geo:GSE2109) and Kao Breast dataset (https://identifiers.org/geo:GSE20685) are all available at the NCBI Gene Expression Omnibus (GEO) repository. Additional datasets supporting Figs. 3, 4, and 5 in this article, are available from the corresponding author on reasonable request. Uncropped blots are available as part of the supplementary information. The data generated and analyzed during this study are described in the following data record: https://doi.org/10.6084/m9.figshare.8276132.^31^

## Supplementary information

**Supplementary information** accompanies the paper on the *npj Breast Cancer* website (10.1038/s41523-019-0118-6).
