# Supplementary material for: The phosphatase PPM1A inhibits triple negative breast cancer growth by blocking cell cycle progression
Source: NPJ Breast Cancer. 2019 Jul 26;5:22. doi: 10.1038/s41523-019-0118-6 (PMC6659706; doi:10.1038/s41523-019-0118-6)
Supplement: Supplementary file 1 — Western Blot Data [file 41523_2019_118_MOESM1_ESM.pdf]

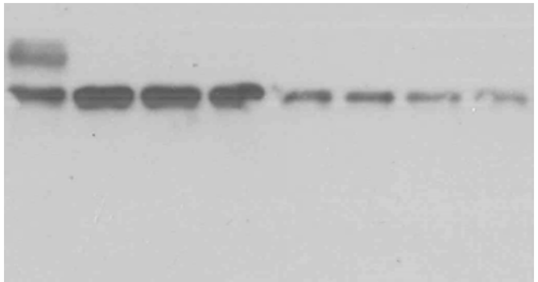

Figure 3A (upper)
WB: PPM1A

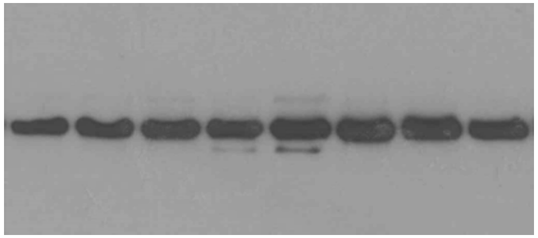

Figure 3A (lower)
WB: Vinculin

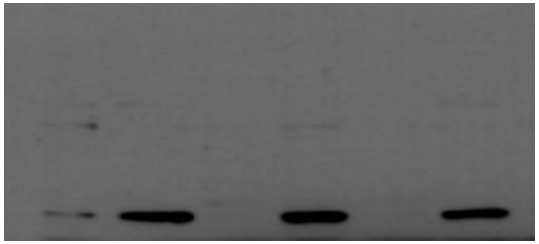

Figure 3B (upper)
WB: PPM1A

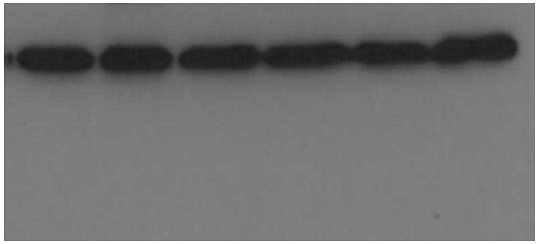

Figure 3B (lower)
WB: Vinculin

Dox - + - +

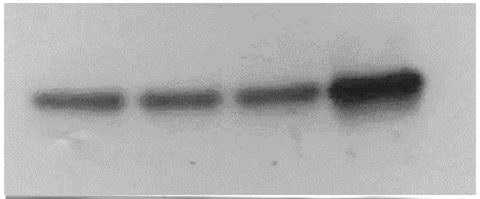

Figure 5D (upper block)
WB: PPM1A

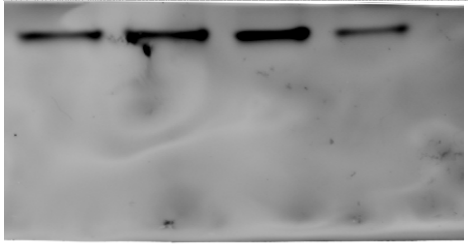

Figure 5D (upper block)
WB: pCDK

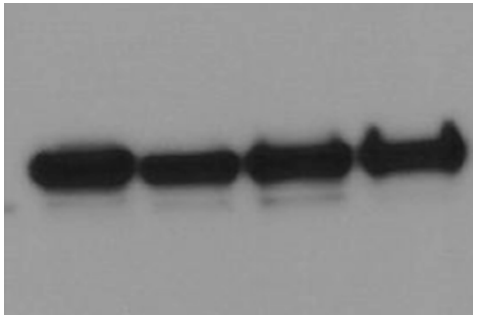

Figure 5D (upper block)
WB: CDK6

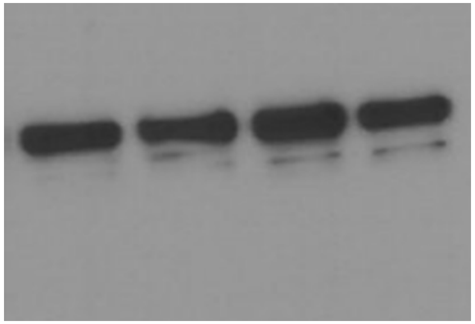

Figure 5D (upper block)
WB: CDK4

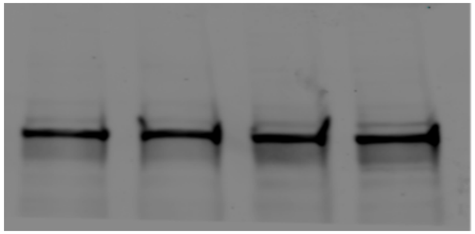

Figure 5D (upper block)
WB: Vinculin

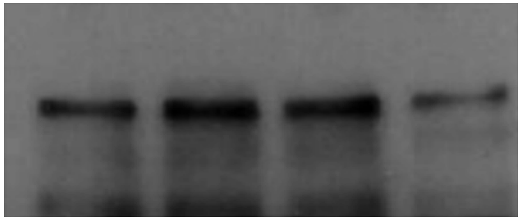

Figure 5D (middle block)
WB: pCDK6

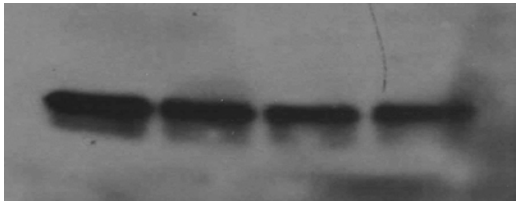

Figure 5D (middle block)
WB: total CDK6

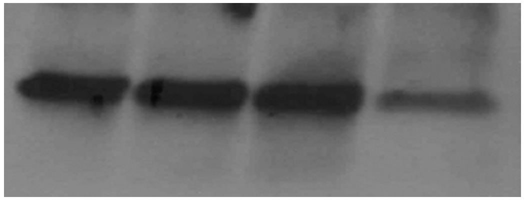

Figure 5D (middle block)
WB: pCDK2

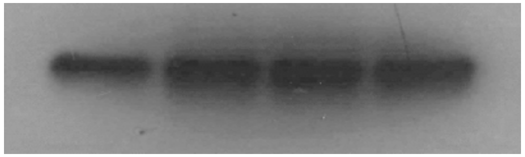

Figure 5D (middle block)
WB: total CDK2

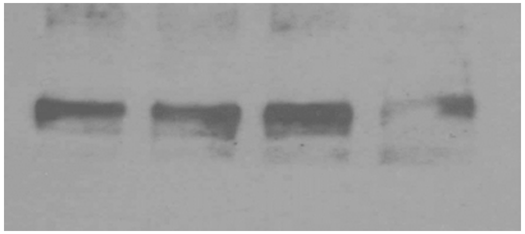

Figure 5D (middle block)
WB: pRb

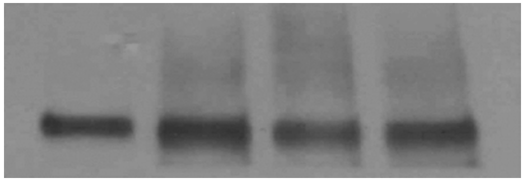

Figure 5D (middle block)
WB: total Rb

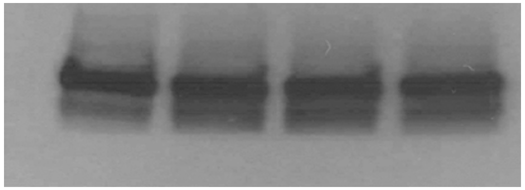

Figure 5D (middle block)
WB: Vinculin

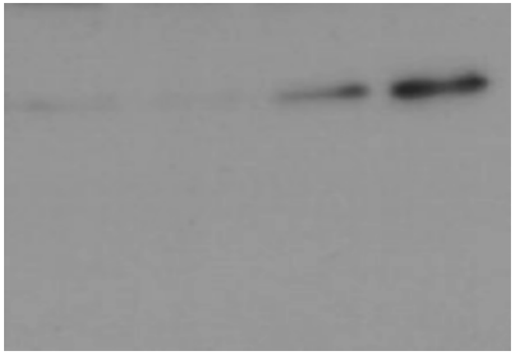

Figure 5D (lower block)
WB: p21

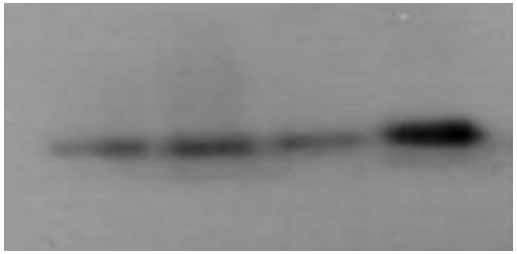

Figure 5D (lower block)
WB: p27

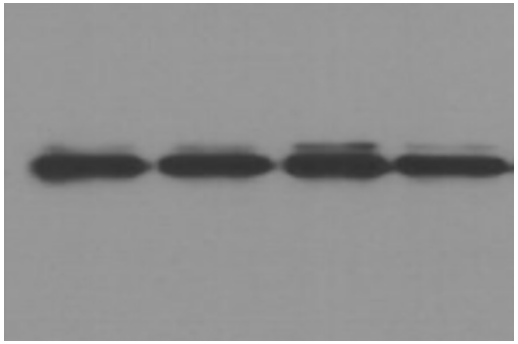

Figure 5D (lower block)
WB: Vinculin

Figure 5D (lower bl
WB: p21

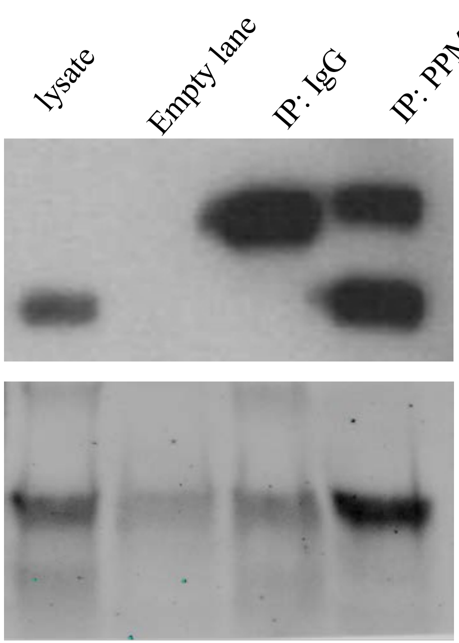

IgG

Figure 5E
WB: PPM1A

Figure 5E
WB: CDK6

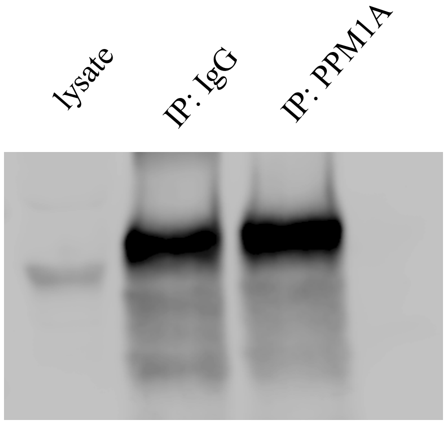

Figure 5E
WB: CDK4

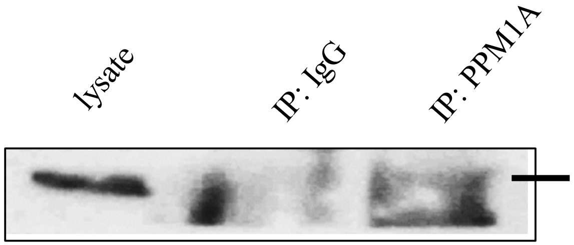

Figure 5E
WB: CDK2
